# Supplementary material for: Simultaneous hyperkinetic movement disorders phenotyping: a cross-cohort pediatric transfer study using routine videos, markerless pose estimation and a tabular foundation model
Source: Front Neurol. 2026 Oct 7;17:1904458. doi: 10.3389/fneur.2026.1904458 (PMC13683199; doi:10.3389/fneur.2026.1904458)
Supplement: Supplementary file 1 [file Table_1.DOCX]

**CODY-SAMP VIDEO PROTOCOL AND INSTRUCTIONS FOR RATERS**

**INSTRUCTION FOR VIDEO PROTOCOL RECORDING** (times are given for information only)

Guidelines for the video recording and for patient instructions are indicated below.

| **REST CONDITION (mainly)** | |
| --- | --- |
| **LYING DOWN - FULL BODY; Camera in “High Position”** | |
| Keepat rest whole body | 30 seconds |
| Deep tendon reflexes (4 limbs) | 30 seconds |
| Plantar cutaneous reflex | 20 seconds |
| Muscle tone examination | 120 seconds |
| Clap test (for reflex myoclonus examination) | 5 seconds |
| Mingazzini maneuver | 20 seconds |
| Heel to shin test bilaterally | 40 seconds |
| **SITTING POSITION, FRONT, UPPER BODY PART; Camera in “Low Position”** | |
| Sitting, arms supine and relaxed on thighs | 30 seconds |
| **POSTURE PART** | |
| **SITTING POSITION, FULL BODY** | |
| Arms and wrists outstretched, pronated | 20 seconds |
| Arms outstretched pronated, wrists extension | 15 seconds |
| Bretteur's maneuver (bent elbows, fingers facing) | 15 seconds |
| Arms and wrists outstretched supinated | 15 seconds |
| Right side head and neck rotation then maintain the posture | 5 seconds |
| Left side head and neck rotation then maintain the posture | 5 seconds |
| Right side head and neck tilt then maintain the posture | 5 seconds |
| Left side head and neck tilt then maintain the posture | 5 seconds |
| Head and neck flexion | 5 seconds |
| Head and neck extension | 5 seconds |
| **ACTION PART** | |
| **SITTING POSITION, FULL BODY** | |
| Alternating finger to nose maneuver | 20 seconds |
| Point to-nose test (physician’s moving finger) right arm | 15 seconds |
| Point-to-nose test (physician’s moving finger) left arm | 15 seconds |
| Right upper limb bradykinesia testing (finger tapping tests, hand opening and closing) | 20 seconds |
| Left upper limb bradykinesia testing (finger tapping tests, hand opening and closing) | 20 seconds |
| Bilateral rapid alternating movements of the forearms horizontally and vertically | 10 seconds |
| **SITTING POSITION, WHOLE BODY** | |
| Right lower limb bradykinesia testing (as foot or leg tapping test) | 20 seconds |
| Left lower limb bradykinesia testing (as or leg tapping test) | 20 seconds |
| Arising from the chair three times, if possible, without support | 20 seconds |
| **SITTING POSITION, UPPER BODY,** *Camera is zooming on the face* | |
| Blinking x 5 | 5 seconds |
| Ask to close eyelids, holding then open them | 5 seconds |
| Mouth open, tongue at rest | 5 seconds |
| Alternating opening and closing mouth movements | 5 seconds |
| Tongue protrusion | 5 seconds |
| Tongue protrusion with lateral movements | 10 seconds |
| Repeat a sentence | 10 seconds |
| Maintaining a vowel sound (ex. eeeeeeeee) | 5 seconds |
| Writing down a sentence or a few words in younger children | 10 seconds |
| Spiral drawing, right hand | 5 seconds |
| Spiral drawing, left hand | 5 seconds |
| Maintain then pour a glass of water with right hand | 10 seconds |
| Maintain then pour a glass of water with left hand | 10 seconds |
| **POSTURE and ACTION: STANDING POSTION, IN FRONT OF HE CAMERA, WHOLE BODY**  *Camera in high position* | |
| Standing posture, still, arms alongside of the body, eyes open | 10 seconds |
| Standing posture, outstretched arms in front, eyes closed | 5 seconds |
| Body rotation of 90° to the right, left side view | 5 seconds |
| Rotation of 90° more, back view standing posture | 5 seconds |
| Rotation of 90° more, right side view | 5 seconds |
| **GAIT, WHOLE BODY** | |
| Natural gait straight forward and turning both sides | 30 seconds |
| Heel to toe walking test (tandem gait, 10 steps) | 20 seconds |
| Tiptoe walk | 10 seconds |
| Backward walk | 15 seconds |

*TOTAL TIME OF VIDEO PROTOCOL: approx.12 minutes and 40 seconds.*

**VIDEO RECORDING GUIDELINES**

1. Patient Consent

Before beginning any video-recorded examination, informed consent must be obtained from the patient or their legal guardian Patient should be informed about the purpose of the recording, how it will be stored and used.

2. Camera Setup

All video recordings should be conducted using a fixed camera or smartphone mounted on a tripod.

Technical specifications:

- Resolution: Minimum 1080p (1920×1080 pixels)

- Frame rate: Minimum 30 frames per second

- Audio: Clear recording capability for speech assessment

- Lighting: Uniform illumination without shadows on the patient

Camera positioning:

The camera distance and height will vary depending on the patient's position as detailed in the protocol.

Two distinct camera positions are required:

- "High position": for standing/ gait Camera positioned above the patient tilted 45° downward and 1 meter away

- "Low position" for seated tasks positioned at least 2 meters away from the chair adjusted to patients height 90° angle of the ground (approximately chest level of a standing examiner)

Note: For gait assessment, increase distance to 4 meters to capture full walking path.

3. Patient Clothing Requirements

For optimal clinical observation the patient must be barefoot (no shoes or socks). Short sleeves would be recommended for better visualization of the limbs.

4. Required Equipment

The following items must be available in the examination room:

- Examination table (for supine assessment)

- Sturdy chair without armrests (for seated assessment)

- Desk or table (for writing and pouring tasks)

- Reflex hammer

- Two transparent glasses

- Pitcher of water

- Pen

- Blank paper sheet (for writing and drawing tasks)

**PATIENT INSTRUCTIONS DURING THE CLINICAL EXAMINATION**

Please lie down

- You can stay relaxed, without moving, at rest, for 30 seconds
- I will now test your arm and leg deep tendon reflexes
- I will gently touch the sole of your foot on both sides to check a reflex
- Let your arms and legs and neck stay loose. I will check your muscle tone by gently moving each body segment.
- *The examiner does not warn the patient and claps their hands loudly to assess whether myoclonus occurs in response to the auditory stimulus*
- Please lie on your back. Raise both legs like you're riding a bike and keep on holding them up for 20 seconds (Mingazzini test). This allows us to test their strength
- Slide your heel along the opposite leg, from knee to the foot several times (both sides).

Please come and sit down on this chair.

First with your hands resting on your thighs, arms relaxed.

- Now, you can stretch your arms forward, palms facing down; hold the position
- Same position, but lift your wrists up
- Bend your elbows, bring your hands in front of your chest with index fingers aligned
- Stretch your arms forward again, palms facing up; hold the position

Now relax your arms

- Turn your head to the right and maintain this posture
- Turn your head to the left and maintain this posture
- Tilt your head to the right and hold the posture
- Tilt your head to the left and hold the posture
- Bend your head forward (chin to chest)
- Lift your head up and look at the ceiling

I am checking now how you perform a few actions

- Touch your nose with your index finger several times bilaterally, alternating your arms
- Touch your nose with you right index, then my finger, and repeat it while I am moving my finger
- Now do the same with your left index finger
- Tap quickly you thumb with your right index finger 10 times and/or close and open 10 times your right hand
- Repeat it with your left hand.
- Please turn your hands together horizontally and vertically several times
- Tap the floor with your right toes or/and the full leg several times, as fast as possible ten times.
- Tap the floor with your right toes or/and the full leg several times, as fast as possible ten times.
- Stand up from the chair three times, if possible, without using your hands.

I am now zooming your face, please remain sitting.

- Blink five times.
- Close your eyes tightly, hold, then open them.
- Open your mouth and keep your tongue inside your mouth
- Open and close your mouth several times
- Stick out your tongue out and maintain this it for a few seconds
- Move your tongue side to side.
- Please repeat this sentence after me “...” (it can be for example “I am in (name of the town) and the weather is good”, or the days of the week).
- Hold a vowel sound like “eeeeeeee.”

Now we will focus on your arms. Here you have a pen and a paper sheet

- Please write a short sentence (it can be the one that you said just before; please do not write your name for confidential issues).
- Draw a spiral with your right hand without letting your wrist touch the paper sheet
- Please repeat the same task with your left hand. (* Inform whether the patient is right-handed or not)
- Hold this glass of water with your right hand, then pour it in the empty glass
- Please repeat with your left hand

We are now close to the end of the clinical examination. We will now assess standing position and gait

- Please stand still with your arms by your sides, eyes open
- Stretch your arms forward and close your eyes
- Relax your arms and turn your body to the right (lateral view)
- Turn again (back to the camera)
- Turn again (other side, lateral view).
- Walk straight forward, then turn around.
- Walk heel-to-toe on a straight line for 10 steps just like me (please demonstrate)
- Walk on your tiptoes.
- Walk backward.

The examination is now complete. Thank you for your collaboration. Now the camera is turned off.
