# Supplementary material for: Simultaneous hyperkinetic movement disorders phenotyping: a cross-cohort pediatric transfer study using routine videos, markerless pose estimation and a tabular foundation model
Source: Front Neurol. 2026 Oct 7;17:1904458. doi: 10.3389/fneur.2026.1904458 (PMC13683199; doi:10.3389/fneur.2026.1904458)
Supplement: Supplementary file 2 [file Table_2.DOCX]

Supplementary Material S2: Detailed methods and mathematical formulation

Video-based phenotyping using markerless pose estimation and a tabular foundation model

# 1 Detailed methods and mathematical formulation

This supplement provides the detailed technical and mathematical description of the pipeline summarized in the main text. It is organized as follows: 1.1 fixes notation; 1.2 describes pose estimation; 1.3 describes time-series pre-processing; 1.4 derives posture-centric signals; 1.5 defines the windowing scheme; 1.6 specifies the per-window kinematic descriptors; 1.7 describes signal-level normalization; 1.8 describes the TabICLv2 backbone; 1.9 describes class-imbalance handling; 1.10 describes cross-validation; 1.11 describes patient-level aggregation; 1.12 describes threshold tuning on the development cohort; 1.13 describes the dataset-specific local calibration; 1.14 formalizes the reference-label constructions; 1.15 specifies the evaluation metrics; 1.16 reports implementation details.

## 1.1 Notation and conventions

Let $\mathcal{V}$ denote the set of clinical video recordings. For each video $v\mathcal{\in V}$, let $T_{v}$ be the number of analyzed frames and $f_{v}$ the frame rate (frames per second, fps). Throughout the pipeline we assume $f_{v}=30$ fps unless otherwise specified. We denote:

- $\mathcal{P}$: the set of patients (and controls). For each patient $p\mathcal{\in P}$, $V_{p}\mathcal{\subseteq V}$ are the videos collected from $p$.
- $\mathcal{L=\{}\text{Dystonia},\text{Tremor},\text{Myoclonus},\text{Chorea},\text{Athetosis},\text{Ballismus},\text{Stereotypies},\text{Tics}\}$: the eight target hyperkinetic phenomenologies, with $\left| \mathcal{L} \right|=L=8$.
- $\mathcal{K=\{}1,\ldots,17\}$: the indices of the 17 anatomical keypoints in the COCO topology.
- $\mathcal{S}$: the set of analyzed signals (per-keypoint $x$, $y$, distance, plus optional posture-centric signals).
- $\mathcal{W}_{p}$: the set of analyzed temporal windows for patient $p$.

For a real-valued vector $\mathbf{x}=\left( x_{1},\ldots,x_{n} \right)$, we use $\bar{\mathbf{x}}$, $\mathrm{med}\left( \mathbf{x} \right)$, $\mathrm{std}\left( \mathbf{x} \right)$, $\mathrm{IQR}\left( \mathbf{x} \right)$ and $Q_{q}\left( \mathbf{x} \right)$ for the empirical mean, median, standard deviation, interquartile range and the empirical $q$-th quantile, respectively.

## 1.2 Pose estimation

Each frame $t\in\{1,\ldots,T_{v}\}$ of a video $v$ is processed with the YOLOv8x-Pose-P6 detector (Ultralytics), pretrained on the COCO Keypoints dataset for 17-keypoint human pose estimation. For frame $t$, the detector outputs a set of candidate persons; when at least one person is detected, we take the highest-confidence detection and store its 17 keypoints. For each keypoint $k\mathcal{\in K}$, the detector returns normalized coordinates $\left( \tilde{x}_{t,k},\tilde{y}_{t,k} \right)\in\left[ 0,1 \right]^{2}$, which are converted to pixel coordinates using the video frame dimensions $\left( W,H \right)$:

$$x_{t,k}=round\left( \tilde{x}_{t,k}\cdot W \right), y_{t,k}=round\left( \tilde{y}_{t,k}\cdot H \right).$$

If no person is detected for frame $t$, all $\left( x_{t,k},y_{t,k} \right)$ are recorded as missing.

For each keypoint $k$, an absolute distance to the image origin is also computed:

$$d_{t,k}=\sqrt{x_{t,k}^{2}+y_{t,k}^{2}}.$$

The output for video $v$ is therefore a structured time series with frame index $t$, timestamps $\tau_{t}=t/f_{v}$, and for each keypoint $k$ three time-aligned channels: $x_{t,k}$, $y_{t,k}$ and $d_{t,k}$. The 17 keypoints follow the COCO topology (nose, left/right eye, left/right ear, left/right shoulder, left/right elbow, left/right wrist, left/right hip, left/right knee, left/right ankle).

## 1.3 Time-series pre-processing for inference

Inference videos do not carry explicit temporal annotations. Their pose-derived time series may contain frames with no detection or with sparse keypoints due to occlusion, pose ambiguity or low recording quality. The pre-processing pipeline therefore performs three operations:

**(i) Drop fully empty rows.** A row $t$ is removed if all coordinate columns are missing:

$$\forall k\mathcal{\in K:}x_{t,k}=NaN \wedge y_{t,k}=NaN.$$

**(ii) Optionally drop sparse rows.** Let $m_{t}$ denote the per-row missingness rate over coordinate columns:

$$m_{t}=\frac{1}{2\left| \mathcal{K} \right|}\sum_{k\mathcal{\in K}} \left( \mathbf{1}\left[ x_{t,k}=NaN \right]+\mathbf{1}\left[ y_{t,k}=NaN \right] \right).$$

A row is dropped if $m_{t}\geq\rho$, where $\rho\in\left[ 0,1 \right]$ is a configurable sparsity threshold.

**(iii) Reconstruct distance signals.** When the per-keypoint distance signal $d_{t,k}$ is missing or absent in the input file, it is recomputed from the (cleaned) coordinates as a per-frame displacement:

$$\Delta x_{t,k}=x_{t,k}-x_{t-1,k}, \Delta y_{t,k}=y_{t,k}-y_{t-1,k},$$

$$d_{t,k}^{\text{disp}}=\sqrt{\left( \Delta x_{t,k} \right)^{2}+\left( \Delta y_{t,k} \right)^{2}},$$

with $d_{t,k}^{\text{disp}}=NaN$ whenever any of $x_{t,k}$, $y_{t,k}$, $x_{t-1,k}$, $y_{t-1,k}$ is missing or whenever the inter-frame interval $\tau_{t}-\tau_{t-1}$ exceeds a tolerance $\tau_{\max}=2/f_{v}$ (gap detection). The pipeline also produces a data-quality report containing: the number of rows before/after each cleaning step, the fraction of dropped rows, the per-row coordinate missing rate after cleaning, and the number of distance values reconstructed.

## 1.4 Posture-centric derived signals

When sufficient trunk landmarks are available, additional posture-related signals are computed at the frame level. Let $\mathbf{p}_{LS},\mathbf{p}_{RS},\mathbf{p}_{LH},\mathbf{p}_{RH}\in\mathbb{R}^{2}$ denote the left/right shoulder and left/right hip 2D positions at a given frame. Define the midpoints:

$$\mathbf{m}_{S}=1/2\left( \mathbf{p}_{LS}+\mathbf{p}_{RS} \right), \mathbf{m}_{H}=1/2\left( \mathbf{p}_{LH}+\mathbf{p}_{RH} \right), \mathbf{c}_{T}=1/2\left( \mathbf{m}_{S}+\mathbf{m}_{H} \right).$$

The shoulder width (used as an approximate body-scale unit) is:

$$w_{S}=\parallel\mathbf{p}_{RS}-\mathbf{p}_{LS}\parallel_{2}.$$

Shoulder, hip and trunk angles are computed from the relevant vectors:

$$\theta_{S}=\arctan_{2}\left( y_{RS}-y_{LS},x_{RS}-x_{LS} \right),$$

$$\theta_{H}=\arctan_{2}\left( y_{RH}-y_{LH},x_{RH}-x_{LH} \right),$$

$$\theta_{T}=\arctan_{2}\left( y_{m_{S}}-y_{m_{H}},x_{m_{S}}-x_{m_{H}} \right).$$

All angles are wrapped to $(-\pi,\pi]$ via $\theta\mapsto mod\left( \theta+\pi,2\pi\right)-\pi$. Trunk torsion is the angular difference between shoulder and hip lines:

$$\theta_{\text{tors}}=wrap\left( \theta_{S}-\theta_{H} \right).$$

Two scale-normalized vertical asymmetries are computed:

$$\Delta y_{S}=\left( y_{RS}-y_{LS} \right)/w_{S}, \Delta y_{H}=\left( y_{RH}-y_{LH} \right)/w_{S}.$$

Two scale-normalized trunk biases relative to the mid-hip point:

$$b_{x}=\left( c_{T,x}-m_{H,x} \right)/w_{S}, b_{y}=\left( c_{T,y}-m_{H,y} \right)/w_{S}.$$

When neck and nose landmarks are also available, an optional head angle is derived as $\theta_{\text{head}}=\arctan_{2}\left( y_{\text{nose}}-y_{\text{neck}},x_{\text{nose}}-x_{\text{neck}} \right)$, then wrapped to $(-\pi,\pi]$. These nine to ten posture-centric signals (shoulder_width, shoulder_angle, hip_angle, torsion, trunk_angle, shoulder_height_diff, hip_height_diff, trunk_bias_x, trunk_bias_y, optional head_angle) are appended to the per-keypoint signals and treated identically downstream.

## 1.5 Window construction

Two windowing regimes are used.

**Annotated windows (development cohort).** Each video carries clinician-defined start and end times $\left( \mathrm{From}_{w},\mathrm{To}_{w} \right)$, both in seconds. The resulting analysis windows are explicitly aligned to expert-rated segments. The label vector $\mathbf{y}_{w}\in\{0,1,2{\}}^{L}$ for window $w$ is the set of per-phenomenology codes assigned by the rater (0 = absent, 1 = present, 2 = uncertain).

**Sliding windows (external cohorts).** When no temporal annotation is available, a fixed-size sliding window of length $W=300$ frames and stride $S=150$ frames is applied along the cleaned time-series, corresponding at $f_{v}=30$ fps to $W/f_{v}=10$ s windows with $S/f_{v}=5$ s stride and 50% overlap. For a series of $T$ valid frames, the set of windows is

$$\mathcal{W=}\left\{ \left[ s,s+W-1 \right]:s\in\{0,S,2S,\ldots\}, s+W-1\leq T-1 \right\}.$$

If $T<W$, a single window covering the whole series is used. No temporal label is associated with these windows; they are scored against patient-level reference labels at evaluation time (1.14).

## 1.6 Per-window per-signal kinematic descriptors

Let $\mathbf{s}_{w}=\left( s_{1},\ldots,s_{n} \right)$ denote a single signal restricted to window $w$, with non-missing values only. For each pair (signal, window) the pipeline computes 19 primary descriptors covering distributional, temporal, spectral, distribution-based and complexity domains. We list them with their definitions.

### 1.6.1 Distributional descriptors

- **Mean**: $\bar{s}=\frac{1}{n}\sum_{i=1}^{n} s_{i}$.
- **Variance**: $\mathrm{var}\left( \mathbf{s} \right)=\frac{1}{n}\sum_{i=1}^{n} \left( s_{i}-\bar{s} \right)^{2}$.
- **Standard deviation** (sample, $n>1$): $\mathrm{std}\left( \mathbf{s} \right)=\sqrt{\frac{1}{n-1}\sum_{i=1}^{n} \left( s_{i}-\bar{s} \right)^{2}}$.
- **Min, Max, Range**: $\min_{i}s_{i}$, $\max_{i}s_{i}$, $\mathrm{range}\left( \mathbf{s} \right)=\max_{i}s_{i}-\min_{i}s_{i}$.
- **Median**: $\mathrm{med}\left( \mathbf{s} \right)$.
- **Skewness** (sample, bias-corrected): $g_{1}=\frac{n^{2}}{\left( n-1 \right)\left( n-2 \right)}\cdot\frac{1}{n}\sum_{i} (\frac{s_{i}-\bar{s}}{\sigma})^{3}$, with $\sigma=std\left( \mathbf{s} \right)$.
- **Kurtosis** (sample excess, bias-corrected): $g_{2}=\left[ \frac{1}{n}\sum_{i} \left( s_{i}-\bar{s} \right)^{4}/\sigma^{4} \right]-3$ (Fisher form).
- **Interquartile range**: $\mathrm{IQR}\left( \mathbf{s} \right)=Q_{0.75}\left( \mathbf{s} \right)-Q_{0.25}\left( \mathbf{s} \right)$.

### 1.6.2 Temporal descriptors

- **Slope**. Let $\boldsymbol{\beta}\in\mathbb{R}^{2}$ be the OLS coefficients of the simple linear regression $s_{i}=\beta_{0}+\beta_{1}\cdot i+\varepsilon_{i}$. The slope feature is $\beta_{1}$.
- **Energy**: $E\left( \mathbf{s} \right)=\sum_{i=1}^{n} s_{i}^{2}$.
- **Mean absolute acceleration**: with second-order finite differences $a_{i}=s_{i+1}-2s_{i}+s_{i-1}$ for $i=2,\ldots,n-1$,

$$abs\_accel\_mean\left( \mathbf{s} \right)=\frac{1}{n-2}\sum_{i=2}^{n-1} \left| a_{i} \right|.$$

- **Zero crossings of the first difference**. Let $\delta_{i}=s_{i+1}-s_{i}$. The feature is the number of consecutive sign reversals:

$$\mathrm{ZC}_{\delta}\left( \mathbf{s} \right)=\sum_{i=1}^{n-2} \mathbf{1}\left[ \delta_{i}\cdot\delta_{i+1}<0 \right].$$

- This counts changes in the direction of motion within the window.

### 1.6.3 Spectral descriptors

Let $\hat{\mathbf{s}}=rFFT\left( \mathbf{s}-\bar{s} \right)$ denote the one-sided discrete Fourier transform of the centered signal, with associated frequency bins $f_{k}=k\cdot f_{v}/n$ for $k=0,\ldots,\lfloor n/2\rfloor$. The pipeline computes:

- **Dominant peak frequency**: $f^{*}=f_{k^{*}}$, with $k^{*}=arg\max_{k\geq1}\left| \hat{s}_{k} \right|$ (the DC term is excluded).
- **Dominant peak amplitude**: $A^{*}=\left| \hat{s}_{k^{*}} \right|$.

These two descriptors summarize the dominant rhythmic component of the signal within the window.

### 1.6.4 Distribution-based entropy

The histogram-based Shannon entropy is computed from $B=20$ equal-width bins covering the observed range of $\mathbf{s}$:

$$H_{\text{hist}}\left( \mathbf{s} \right)=-\sum_{b=1}^{B} \pi_{b}\log\pi_{b}, \pi_{b}=\frac{n_{b}+\varepsilon}{N+B\varepsilon},$$

where $n_{b}$ is the number of values falling in bin $b$, $N=\sum_{b} n_{b}$ and $\varepsilon={10}^{-12}$ is a numerical floor.

### 1.6.5 Complexity descriptors

- **Higuchi fractal dimension** (Higuchi 1988). For each $k\in\{1,\ldots,k_{\max}\}$ with $k_{\max}=6$, and for each starting offset $m\in\{0,\ldots,k-1\}$, define the curve length

$$L_{m}\left( k \right)=\frac{n-1}{k \lfloor\left( n-m \right)/k\rfloor}\sum_{j=1}^{\lfloor\left( n-m-1 \right)/k\rfloor} |s_{m+jk}-s_{m+\left( j-1 \right)k}|.$$

Let $\bar{L}\left( k \right)=\frac{1}{k}\sum_{m=0}^{k-1} L_{m}\left( k \right)$. The Higuchi fractal dimension is the negative slope obtained by linear regression of $\log\bar{L}\left( k \right)$ against $\log\left( 1/k \right)$ across $k=1,\ldots,k_{\max}$.

- **Permutation entropy** (Bandt & Pompe 2002). For embedding order $D=3$ and delay $\Delta=1$, define the embedded vectors $\mathbf{v}_{i}=\left( s_{i},s_{i+\Delta},s_{i+2\Delta} \right)$ for $i=1,\ldots,n-2\Delta$. Each $\mathbf{v}_{i}$ is mapped to its ordinal pattern $\pi\left( \mathbf{v}_{i} \right)\in\mathfrak{S}_{D}$ (permutation that sorts the entries in ascending order). Let $p_{\pi}$ be the empirical frequency of pattern $\pi$. The normalized permutation entropy is

$$H_{\text{PE}}\left( \mathbf{s} \right)=-\frac{1}{\log D!}\sum_{\pi\in\mathfrak{S}_{D}} p_{\pi}\log p_{\pi}\in\left[ 0,1 \right].$$

A value close to 1 indicates a near-uniform distribution of ordinal patterns (high disorder); a value close to 0 indicates strong ordinal structure (low disorder).

### 1.6.6 Recap and feature naming

For a signal column named c (e.g., left_wrist_x, posture__torsion), each descriptor named d (e.g., mean, higuchi_fd) yields a feature column named f__c__d. With $\left| \mathcal{S} \right|$ signals and 19 descriptors, the per-window feature vector has dimension $19\cdot\left| \mathcal{S} \right|$. **Table S2.1** summarizes the 19 descriptors.

| Descriptor | Domain | Formula / definition |
| --- | --- | --- |
| mean | distribution | $\bar{s}$ |
| std | distribution | $\sqrt{\frac{1}{n-1}\sum\left( s_{i}-\bar{s} \right)^{2}}$ |
| var | distribution | $\frac{1}{n}\sum\left( s_{i}-\bar{s} \right)^{2}$ |
| min | distribution | $\min_{i}s_{i}$ |
| max | distribution | $\max_{i}s_{i}$ |
| median | distribution | $\mathrm{med}\left( \mathbf{s} \right)$ |
| range | distribution | $max-min$ |
| skew | distribution | bias-corrected sample skewness |
| kurtosis | distribution | bias-corrected sample excess kurtosis (Fisher) |
| iqr | distribution | $Q_{0.75}-Q_{0.25}$ |
| slope | temporal | OLS slope of $s_{i}$ vs $i$ |
| energy | temporal | $\sum s_{i}^{2}$ |
| abs_accel_mean | temporal | mean $\left\vert s_{i+1}-2s_{i}+s_{i-1} \right\vert$ |
| zero_crossings_delta | temporal | sign reversals of first difference |
| fft_peak_freq | spectral | argmax frequency of $\left\vert\mathrm{rFFT}\left( \mathbf{s}-\bar{s} \right) \right\vert$ |
| fft_peak_amp | spectral | corresponding magnitude |
| entropy | distribution-based | Shannon entropy of 20-bin histogram |
| higuchi_fd | complexity | Higuchi fractal dimension, $k_{\max}=6$ |
| perm_entropy | complexity | normalized permutation entropy, $D=3,\Delta=1$ |

**Table S2.1.** Per-signal per-window kinematic descriptors used as inputs to the tabular foundation model.

## 1.7 Robust normalization

To reduce sensitivity to scale differences and outliers between recordings, each signal can optionally be normalized at the window level *before* feature computation. The robust transformation is

$$\tilde{s}_{i}=\frac{s_{i}-med\left( \mathbf{s} \right)}{\sigma_{\mathrm{rob}}\left( \mathbf{s} \right)}, \sigma_{\mathrm{rob}}\left( \mathbf{s} \right)=\left\{ \begin{matrix} \mathrm{IQR}\left( \mathbf{s} \right) & \text{if }\mathrm{IQR}\left( \mathbf{s} \right)>\varepsilon, \\ \mathrm{std}\left( \mathbf{s} \right)+\varepsilon& \text{otherwise}, \end{matrix} \right.$$

with $\varepsilon={10}^{-9}$. This step is applied to every signal independently and is strictly local (per window, per signal); there is no global standardization across recordings.

## 1.8 TabICLv2 backbone

We use TabICLv2 (Qu et al. 2026), a tabular foundation model designed for in-context classification on structured data and pretrained on a large distribution of synthetic and real tabular tasks (Hollmann et al. 2025; Qu et al. 2025). Conceptually, TabICLv2 is a transformer-based learner that, given a labeled context $\left( \mathbf{X}_{\text{ctx}},\mathbf{y}_{\text{ctx}} \right)$ and a query $\mathbf{x}_{q}$, returns $P\left( y_{q}=1\mid\mathbf{x}_{q},\mathbf{X}_{\text{ctx}},\mathbf{y}_{\text{ctx}} \right)$ without any task-specific gradient update at inference time. In this study, TabICLv2 acts as the **shared predictive backbone** that maps a per-window feature vector to a per-window probability of symptom presence.

Because the clinical target is multi-label (a patient may simultaneously exhibit several phenomenologies), prediction is decomposed as $L=8$ independent one-vs-rest binary problems. For each label $\mathcal{l\in L}$, an independent TabICLv2 classifier $g_{\mathcal{l}}$ is fitted to the development cohort, mapping a per-window feature vector $\mathbf{x}$ to a probability $p_{\mathcal{l}}\left( \mathbf{x} \right)=g_{\mathcal{l}}\left( \mathbf{x} \right)\in\left[ 0,1 \right]$. This decomposition is preferred to a single multi-label head because (i) it allows each phenomenology to have its own decision boundary and prevalence profile; (ii) it allows label-specific aggregation and threshold tuning (1.11–1.13); and (iii) it avoids forcing a single shared softmax over phenomenologies that are not mutually exclusive. The hyperparameters used in this study are listed in **Table S2.2**.

| Hyperparameter | Value |
| --- | --- |
| Checkpoint version | tabicl-classifier-v2-20260212.ckpt |
| n_estimators (ensemble size) | 32 |
| softmax_temperature | 0.8 |
| batch_size | 64 |
| use_amp | auto |
| outlier_threshold | 6.0 |
| random_state | 42 |

**Table S2.2.** TabICLv2 hyperparameters used for one-vs-rest training.

## 1.9 S2.9 Class-imbalance handling

For each label $\mathcal{l}$, the development cohort exhibits substantial class imbalance: most windows are negative for any given phenomenology. Three measures are applied.

**(i) Uncertainty-aware exclusion.** Windows with $y_{w}^{\left( \mathcal{l} \right)}=2$ (“uncertain”) are removed from the training set of label $\mathcal{l}$, but retained for the training sets of other labels for which the rater provided $\{0,1\}$. Concretely, for label $\mathcal{l}$ and window $w$, $w$ is retained iff $y_{w}^{\left( \mathcal{l} \right)}\in\{0,1\}$.

**(ii) Stratified negative subsampling with control preservation.** Let $n_{\mathcal{l}}^{+}$ denote the number of positive windows for label $\mathcal{l}$ after step (i). The pipeline targets a maximum negative-to-positive ratio $\rho_{\text{npr}}$ (default $\rho_{\text{npr}}=20$). Define the negative budget $N_{\text{neg}}=min\left( \lceil\rho_{\text{npr}}\cdot n_{\mathcal{l}}^{+}\rceil,n_{\mathcal{l}}^{-} \right)$. Negatives are sampled in two strata:

$$N_{\text{ctrl}}=min\left( \lfloor\alpha\cdot N_{\text{neg}}\rfloor,n_{\text{ctrl}}^{-} \right), N_{\text{pat}}=min\left( N_{\text{neg}}-N_{\text{ctrl}},n_{\text{pat}}^{-} \right),$$

with $\alpha=0.30$, $n_{\text{ctrl}}^{-}$ the number of negative windows from healthy controls and $n_{\text{pat}}^{-}$ the number of negative windows from patients with a different phenomenology. Within each stratum, sampling is uniform without replacement. If $N_{\text{neg}}\geq n_{\mathcal{l}}^{-}$, all negatives are kept. This preserves the most informative non-symptomatic distribution (controls capped to avoid over-representing trivial negatives).

**(iii) Minimum-positive guard.** Labels with fewer than $n_{\min}^{+}=5$ positives after step (i) are skipped during training, on the grounds that a TabICLv2 classifier with so few positives is unlikely to yield stable per-window probabilities.

## 1.10 Cross-validation

Because windows are derived from the same patients, simple random splits would leak information across folds. We use **patient-grouped K-fold cross-validation** (GroupKFold with $K=5$): each patient’s windows are entirely contained within either the training or the validation fold. This yields out-of-fold (OoF) per-window probabilities $p_{\mathcal{l}}^{\text{OoF}}\left( w \right)$ for every window $w$ and every label $\mathcal{l}$, used downstream for patient-level aggregation and threshold tuning (1.11–1.12). Disjointness of the training and validation patient sets is verified for each split.

## 1.11 Patient-level aggregation

For a given patient $p$, label $\mathcal{l}$ and aggregation method $a$, the patient-level score is computed by combining all per-window probabilities $\{p_{\mathcal{l}}\left( w \right):w\in\mathcal{W}_{p}\}$ using $a$. Four candidate aggregations are considered:

$$\mathrm{agg}_{\text{mean}}\left( \mathbf{p} \right)=\frac{1}{\left| \mathbf{p} \right|}\sum_{i} p_{i},$$

$$\mathrm{agg}_{\text{max}}\left( \mathbf{p} \right)=\max_{i}p_{i},$$

$$\mathrm{agg}_{\text{p}q}\left( \mathbf{p} \right)=Q_{q}\left( \mathbf{p} \right) \text{for }q\in\{0.70,0.90,0.95\},$$

$$\mathrm{agg}_{\text{noisy-OR}}\left( \mathbf{p} \right)=1-\prod_{i} \left( 1-p_{i} \right).$$

Mean and quantile-based methods are robust to spurious high-probability windows, max captures rare positive evidence, and noisy-OR models the assumption that a single window is sufficient to declare presence. Because phenomenologies have different temporal expression patterns (e.g., dystonia is typically continuous, myoclonus is intermittent), the aggregation method is selected **per label** rather than fixed globally. Let $a_{\mathcal{l}}^{\star}\in\{\text{mean},\text{max},\text{p70},\text{p90},\text{p95},\text{noisy-OR}\}$ denote the aggregation method selected for label $\mathcal{l}$. The patient-level score is

$$S_{p}^{\left( \mathcal{l} \right)}=\mathrm{agg}_{a_{\mathcal{l}}^{\star}}\left( \{p_{\mathcal{l}}\left( w \right):w\in\mathcal{W}_{p}\} \right).$$

A patient-level prediction is then obtained by thresholding:

$$\hat{y}_{p}^{\left( \mathcal{l} \right)}=\mathbf{1}\left[ S_{p}^{\left( \mathcal{l} \right)}\geq\tau_{\mathcal{l}} \right],$$

with label-specific threshold $\tau_{\mathcal{l}}$.

## 1.12 Threshold and aggregation tuning on the development cohort

Aggregation $\mathbf{a}=\left( a_{\mathcal{l}} \right)_{\mathcal{l\in L}}$ and thresholds $\boldsymbol{\tau}=\left( \tau_{\mathcal{l}} \right)_{\mathcal{l\in L}}$ are jointly tuned on out-of-fold probabilities (1.10) to maximize a regularized patient-level objective.

**Patient-level reference.** For each patient $p$ and label $\mathcal{l}$, the patient-level reference $y_{p}^{\left( \mathcal{l} \right)}\in\{0,1,*\}$ is defined from window labels:

$$y_{p}^{\left( \mathcal{l} \right)}=\left\{ \begin{matrix} 1 & \text{if }\exists w\in\mathcal{W}_{p}:y_{w}^{\left( \mathcal{l} \right)}=1, \\ * & \text{else if }\exists w\in\mathcal{W}_{p}:y_{w}^{\left( \mathcal{l} \right)}=2, \\ 0 & \text{otherwise.} \end{matrix} \right.$$

The “*” symbol denotes “unknown” and excludes the corresponding $\left( p\mathcal{,l} \right)$ pair from scoring at this stage. Let $\mathcal{K}_{p\mathcal{,l}}=\mathbf{1}\left[ y_{p}^{\left( \mathcal{l} \right)}\neq* \right]$ denote the known mask.

**Multi-label objective.** Let $\mathbf{Y},\hat{\mathbf{Y}}\in\{0,1{\}}^{\left| \mathcal{P} \right|\times L}$ be the true and predicted patient-level label matrices. The per-patient objective $J\left( \mathbf{Y},\hat{\mathbf{Y}} \right)$ used for tuning is one of:

$$J_{\text{Jaccard}}=\frac{1}{\left| \mathcal{P} \right|}\sum_{p} \frac{\sum_{\mathcal{l:}\mathcal{K}_{p\mathcal{,l}}=1} \mathbf{1}\left[ y_{p}^{\left( \mathcal{l} \right)}=1\wedge\hat{y}_{p}^{\left( \mathcal{l} \right)}=1 \right]}{\max\left( 1,\sum_{\mathcal{l:}\mathcal{K}_{p\mathcal{,l}}=1} \mathbf{1}\left[ y_{p}^{\left( \mathcal{l} \right)}=1\vee\hat{y}_{p}^{\left( \mathcal{l} \right)}=1 \right] \right)},$$

$$J_{\text{exact}}=\frac{1}{\left| \mathcal{P} \right|}\sum_{p} \mathbf{1}\left[ \hat{y}_{p}^{\left( \mathcal{l} \right)}=y_{p}^{\left( \mathcal{l} \right)} \mathcal{\forall l}\text{ with }\mathcal{K}_{p\mathcal{,l}}=1 \right],$$

$$J_{\text{hamming}}=\frac{1}{\left| \mathcal{P} \right|}\sum_{p} \frac{1}{\sum_{\mathcal{l}} \mathcal{K}_{p\mathcal{,l}}}\sum_{\mathcal{l:}\mathcal{K}_{p\mathcal{,l}}=1} \mathbf{1}\left[ \hat{y}_{p}^{\left( \mathcal{l} \right)}=y_{p}^{\left( \mathcal{l} \right)} \right].$$

The default tuning objective is $J_{\text{Jaccard}}$.

**Penalized global score.** To control aggressive positive calling and conservative behavior in healthy controls, the tuned objective is

$$\mathcal{J}_{\text{tune}}\left( \mathbf{a},\boldsymbol{\tau} \right)=J\left( \mathbf{Y},\hat{\mathbf{Y}} \right)-\lambda_{\text{pred}}\cdot\bar{k}\left( \hat{\mathbf{Y}} \right)-\mu_{\text{ctrl}}\cdot c\left( \hat{\mathbf{Y}} \right),$$

with

$$\bar{k}\left( \hat{\mathbf{Y}} \right)=\frac{1}{\left| \mathcal{P} \right|}\sum_{p} \sum_{\mathcal{l}} \hat{y}_{p}^{\left( \mathcal{l} \right)}, c\left( \hat{\mathbf{Y}} \right)=\frac{1}{\left| \mathcal{P}_{\text{ctrl}} \right|}\sum_{p\in\mathcal{P}_{\text{ctrl}}} \mathbf{1}\left[ \sum_{\mathcal{l}} \hat{y}_{p}^{\left( \mathcal{l} \right)}\geq1 \right],$$

$\lambda_{\text{pred}}=0.03$, $\mu_{\text{ctrl}}=0.30$. The first penalty discourages excessive positive predictions; the second strongly discourages any positive label in healthy controls.

**Optimization.** For each label $\mathcal{l}$, the search space is $a_{\mathcal{l}}\mathcal{\in A}$ and $\tau_{\mathcal{l}}\in\mathcal{T}_{\mathcal{l}}$, where $\mathcal{A=\{}\text{p90},\text{p95},\text{max},\text{noisy-OR}\}$ and $\mathcal{T}_{\mathcal{l}}$ is a per-label grid restricted by clinically motivated lower bounds:

$$\mathcal{T}_{\mathcal{l}}\supseteq\{0.05,0.10,0.15,0.20,0.25,0.30,0.35,0.40,0.45,0.50,0.55,0.60,0.65,0.70,0.75\}.$$

Per-label minimum thresholds are imposed as soft floors to avoid trivial overcalling: $\tau_{\text{Tremor}}\geq0.20$, $\tau_{\text{Tics}}\geq0.15$, $\tau_{\text{Ballismus}}\geq0.25$. Optimization proceeds by **block coordinate descent** over labels: at each iteration, for each label $\mathcal{l}$, we replace $\left( a_{\mathcal{l}},\tau_{\mathcal{l}} \right)$ by the pair maximizing $\mathcal{J}_{\text{tune}}$ while keeping all other labels fixed. Iteration stops when no label improves $\mathcal{J}_{\text{tune}}$ (or after a maximum of $T_{\text{cd}}=2$ sweeps). The result is a global aggregation map $\mathbf{a}^{\star}$ and threshold vector $\boldsymbol{\tau}^{\star}$ stored as agg_map.best.json and thresholds.best.json and used as the **default deployment configuration** for any new external dataset.

## 1.13 Local calibration of the decision layer

For each external dataset $\mathcal{D}$, an optional local calibration step adjusts $\left( \mathbf{a},\boldsymbol{\tau} \right)$ on a small labeled subset $\mathcal{C}_{\mathcal{D}}\mathcal{\subset D}$, **without retraining** the per-label TabICLv2 backbones $g_{\mathcal{l}}$. The shared backbone is therefore preserved across cohorts, while the final decision layer absorbs cohort-specific differences in prevalence, annotation conventions and acquisition conditions.

**Calibration sets.** Calibration sets used in this study were:

- *Dataset_2*: $\mathcal{C}_{2}=\{P_{3},P_{5},P_{8},P_{10},P_{11}\}$ ($n=5$ pediatric patients). These five patients were selected by the movement-disorder specialists as representative of the cohort’s phenotypic spectrum; the remaining seven patients were held out and used exclusively for primary external evaluation, while performance on the calibration subset itself was reported separately to gauge overfitting. The uncalibrated baseline reported in the results corresponds to a generic decision rule (a uniform 0.5 threshold with p95 aggregation applied to every label), whereas the calibrated deployment uses the locally fitted per-label aggregation and thresholds.

**Patient-level aggregated probabilities.** Using the OoF probabilities (or external inference probabilities) on $\mathcal{C}_{\mathcal{D}}$, a per-label per-patient cache is precomputed:

$$P_{\mathcal{D}}\left[ \mathcal{l,}a,p \right]=\mathrm{agg}_{a}\left( \{p_{\mathcal{l}}\left( w \right):w\in\mathcal{W}_{p}\} \right), \forall a\in\mathcal{A}_{\mathcal{D}}, \mathcal{\forall l\in L,} \forall p\in\mathcal{C}_{\mathcal{D}},$$

with $\mathcal{A}_{\mathcal{D}}=\{\text{p70},\text{p90},\text{p95},\text{max}\}$ in the dataset_2 setting.

**Per-patient predictions.** Given $\left( \mathbf{a},\boldsymbol{\tau} \right)$, the prediction matrix is

$$\hat{Y}_{p\mathcal{,l}}=\mathbf{1}\left[ P_{\mathcal{D}}\left[ \mathcal{l,}a_{\mathcal{l}},p \right]\geq\tau_{\mathcal{l}} \right].$$

**Calibration objective.** Local calibration maximizes a Jaccard-based score with two clinically motivated penalties:

$$\mathcal{J}_{\text{cal}}\left( \mathbf{a},\boldsymbol{\tau} \right)=J\left( \mathbf{Y}_{\mathcal{D}},{\hat{\mathbf{Y}}}_{\mathcal{D}} \right)-\pi_{\text{ex}}\cdot\left( R_{\text{ex}}+\mathrm{FP}_{\text{ex}}\left( {\hat{\mathbf{Y}}}_{\mathcal{D}},\mathbf{Y}_{\mathcal{D}} \right) \right)-\pi_{\text{fn}}\cdot R_{\text{fn-key}}\left( {\hat{\mathbf{Y}}}_{\mathcal{D}},\mathbf{Y}_{\mathcal{D}} \right),$$

with the following components.

- $J$ is the multi-label objective (default Jaccard, 1.12).
- $\mathrm{FP}_{\text{ex}}$ is the **excess predicted cardinality** rate, normalized by the number of labels:

$$\mathrm{FP}_{\text{ex}}\left( \hat{\mathbf{Y}},\mathbf{Y} \right)=\frac{1}{L}\cdot\frac{1}{\left| \mathcal{P} \right|}\sum_{p} \max\left( 0,\sum_{\mathcal{l}} \hat{y}_{p}^{\left( \mathcal{l} \right)}-\sum_{\mathcal{l}} y_{p}^{\left( \mathcal{l} \right)} \right).$$

- $R_{\text{ex}}$ is the per-patient activation rate of the **non-target labels** at calibration time. For each label $\mathcal{l'\notin}\mathcal{L}_{\text{tgt}}$, a hard threshold $\tau_{\text{ex}}=0.995$ is applied to suppress activations:

$$R_{\text{ex}}=\sum_{\mathcal{l'\notin}\mathcal{L}_{\text{tgt}}} \frac{1}{\left| \mathcal{C}_{\mathcal{D}} \right|}\sum_{p\in\mathcal{C}_{\mathcal{D}}} \mathbf{1}\left[ P_{\mathcal{D}}\left[ \mathcal{l',}a_{\mathcal{l'}},p \right]\geq\tau_{\text{ex}} \right].$$

- $R_{\text{fn-key}}$ is the **false-negative rate restricted to clinically important labels** $\mathcal{L}_{\text{key}}$ (default $\mathcal{L}_{\text{key}}=\{\text{Dystonia},\text{Myoclonus},\text{Chorea}\}$):

$$R_{\text{fn-key}}\left( \hat{\mathbf{Y}},\mathbf{Y} \right)=\mathrm{mean}_{\mathcal{l\in}\mathcal{L}_{\text{key}}}\frac{\#\{p:y_{p}^{\left( \mathcal{l} \right)}=1,\hat{y}_{p}^{\left( \mathcal{l} \right)}=0\}}{\max\left( 1,\#\{p:y_{p}^{\left( \mathcal{l} \right)}=1\} \right)}.$$

- $\pi_{\text{ex}}=0.35$, $\pi_{\text{fn}}=0.35$ control the relative weight of these penalties.

**Search constraints.** Calibration enforces per-label minimum and maximum thresholds to encode clinical priors:

$$\tau_{\mathcal{l}}\in\left[ \tau_{\mathcal{l}}^{\min},\tau_{\mathcal{l}}^{\max} \right],$$

with defaults

$$\tau_{\text{Tremor}}^{\min}=0.35,\tau_{\text{Tics}}^{\min}=0.35,\tau_{\text{Ballismus}}^{\min}=0.30,\tau_{\text{Stereotypies}}^{\min}=0.25,\tau_{\text{Athetosis}}^{\min}=0.20,$$

$$\tau_{\text{Dystonia}}^{\max}=0.55,\tau_{\text{Myoclonus}}^{\max}=0.50,\tau_{\text{Chorea}}^{\max}=0.55.$$

These bounds prevent the optimizer from collapsing thresholds onto degenerate solutions (e.g., $\tau\approx0$ for low-prevalence labels).

**Optimization.** When the joint search space is small enough (heuristic upper bound: $\left| \mathcal{A} \right|^{L}\cdot\prod_{\mathcal{l}} \left| \mathcal{T}_{\mathcal{l}} \right|\leq2\cdot{10}^{6}$), a full grid search is performed. Otherwise, block coordinate descent is applied as in 1.12, with a maximum of $T_{\text{cd}}=16$ sweeps. The resulting $\left( \mathbf{a}_{\mathcal{D}}^{\star},\boldsymbol{\tau}_{\mathcal{D}}^{\star} \right)$ are stored as agg_map.<dataset>.json and thresholds.<dataset>.json and used at deployment time for that cohort only.

## 1.14 Reference-label constructions

For each external cohort, multiple reference-label schemes were derived from rater-level Excel annotations. The motivation is that single-consensus ground truth oversimplifies inter-rater disagreement, which is itself a clinical signal in movement-disorder phenomenology.

Let $R$ denote the number of raters for the cohort ($R=5$ for the pediatric cohort) and $V_{p}^{\left( \mathcal{l} \right)}=\sum_{r=1}^{R} \mathbf{1}\left[ r\text{ rates }\mathcal{l}\text{ as present in }p \right]$ the number of positive votes for $\left( p\mathcal{,l} \right)$.

**Per-rater labels.** $y_{p,r}^{\left( \mathcal{l} \right)}=1$ iff rater $r$ marked $\mathcal{l}$ as present in $p$, else $0$.

**Majority rule (yes/no).** A label is positive iff $V_{p}^{\left( \mathcal{l} \right)}\geq m_{R}$, with $m_{5}=3$ and $m_{2}=2$:

$$y_{p}^{\left( \mathcal{l} \right),\text{maj}}=\mathbf{1}\left[ V_{p}^{\left( \mathcal{l} \right)}\geq m_{R} \right].$$

For $R=5$, stricter “$k$-of-$R$” rules with $k\in\{4,5\}$ are also used (strict4, strict5):

$$y_{p}^{\left( \mathcal{l} \right),\text{strict-}k}=\mathbf{1}\left[ V_{p}^{\left( \mathcal{l} \right)}\geq k \right].$$

**Soft labels.** The average vote share is reported as

$$\text{soft}_{p}^{\left( \mathcal{l} \right)}=V_{p}^{\left( \mathcal{l} \right)}/R\in\left[ 0,1 \right].$$

**Agreement-aware labels (3 categories).** When a label is supported by a strong majority, it is coded $1$; when it is supported by no rater, it is coded $0$; when intermediate vote counts indicate genuine expert disagreement, it is coded $2$ (uncertain). Concretely:

$$y_{p}^{\left( \mathcal{l} \right),\text{aa}}=\left\{ \begin{matrix} 1 & V_{p}^{\left( \mathcal{l} \right)}\geq4\text{ (}R=5\text{)} \text{or} V_{p}^{\left( \mathcal{l} \right)}=2\text{ (}R=2\text{)}, \\ 0 & V_{p}^{\left( \mathcal{l} \right)}\leq1\text{ (}R=5\text{)} \text{or} V_{p}^{\left( \mathcal{l} \right)}=0\text{ (}R=2\text{)}, \\ 2 & \text{otherwise.} \end{matrix} \right.$$

**Masked labels (configurable).** For dataset 2, three additional schemes are produced by varying the positivity and negativity vote thresholds $\left( k^{+},k^{-} \right)$:

$$y_{p}^{\left( \mathcal{l} \right),\text{masked-}k^{+}k^{-}}=\left\{ \begin{matrix} 1 & V_{p}^{\left( \mathcal{l} \right)}\geq k^{+}, \\ 0 & V_{p}^{\left( \mathcal{l} \right)}\leq k^{-}, \\ 2 & \text{otherwise.} \end{matrix} \right.$$

The schemes used in the manuscript are: masked12 ($k^{+}=3,k^{-}=0$), masked123 ($k^{+}=4,k^{-}=0$), masked1234 ($k^{+}=5,k^{-}=0$), referred to in the main text as Masked (≥3/5; 0/5 negative), Masked (≥4/5; 0/5 negative) and Masked (5/5; 0/5 negative). Labels coded $2$ are excluded from scoring on a per-(patient, label) basis.

For each reference scheme, the corresponding $\left( \mathbf{Y}\mathcal{,K} \right)$ pair (true labels and known mask) feeds into the metrics defined in 1.15.

## 1.15 Evaluation metrics

For a cohort with patient-level true labels $\mathbf{Y}\in\{0,1{\}}^{\left| \mathcal{P} \right|\times L}$, predicted labels $\hat{\mathbf{Y}}\in\{0,1{\}}^{\left| \mathcal{P} \right|\times L}$ and known mask $\mathcal{K\in\{}0,1{\}}^{\left| \mathcal{P} \right|\times L}$, we report:

**Hamming accuracy** (proportion of correctly classified per-(patient, label) pairs over evaluable pairs):

$$HA=\frac{\sum_{p\mathcal{,l}} \mathcal{K}_{p\mathcal{,l}}\cdot\mathbf{1}\left[ \hat{y}_{p}^{\left( \mathcal{l} \right)}=y_{p}^{\left( \mathcal{l} \right)} \right]}{\sum_{p\mathcal{,l}} \mathcal{K}_{p\mathcal{,l}}}.$$

In practice, we report it averaged per patient first to be consistent with patient-level reasoning:

$$\mathrm{HA}_{\text{macro}}=\frac{1}{\left| \mathcal{P} \right|}\sum_{p} \frac{\sum_{\mathcal{l:}\mathcal{K}_{p\mathcal{,l}}=1} \mathbf{1}\left[ \hat{y}_{p}^{\left( \mathcal{l} \right)}=y_{p}^{\left( \mathcal{l} \right)} \right]}{\sum_{\mathcal{l}} \mathcal{K}_{p\mathcal{,l}}}.$$

**Jaccard index** (overlap between predicted and reference positive label sets, per patient, then averaged):

$$J=\frac{1}{\left| \mathcal{P} \right|}\sum_{p} \frac{\sum_{\mathcal{l:}\mathcal{K}_{p\mathcal{,l}}=1} \mathbf{1}\left[ y_{p}^{\left( \mathcal{l} \right)}=1\wedge\hat{y}_{p}^{\left( \mathcal{l} \right)}=1 \right]}{\max\left( 1,\sum_{\mathcal{l:}\mathcal{K}_{p\mathcal{,l}}=1} \mathbf{1}\left[ y_{p}^{\left( \mathcal{l} \right)}=1\vee\hat{y}_{p}^{\left( \mathcal{l} \right)}=1 \right] \right)}.$$

By convention, when both sets are empty, the per-patient term is set to $1$.

**Exact match** (fraction of patients whose full evaluable phenotype profile matches exactly):

$$EM=\frac{1}{\left| \mathcal{P} \right|}\sum_{p} \mathbf{1}\left[ \hat{y}_{p}^{\left( \mathcal{l} \right)}=y_{p}^{\left( \mathcal{l} \right)} \mathcal{\forall l}\text{ with }\mathcal{K}_{p\mathcal{,l}}=1 \right].$$

**Per-label confusion summaries.** For each label $\mathcal{l}$, we count $\mathrm{TP}_{\mathcal{l}},\mathrm{TN}_{\mathcal{l}},\mathrm{FP}_{\mathcal{l}},\mathrm{FN}_{\mathcal{l}}$ over evaluable pairs:

$$\mathrm{TP}_{\mathcal{l}}=\sum_{p} \mathcal{K}_{p\mathcal{,l}}\cdot y_{p}^{\left( \mathcal{l} \right)}\cdot\hat{y}_{p}^{\left( \mathcal{l} \right)}, \mathrm{FP}_{\mathcal{l}}=\sum_{p} \mathcal{K}_{p\mathcal{,l}}\cdot\left( 1-y_{p}^{\left( \mathcal{l} \right)} \right)\cdot\hat{y}_{p}^{\left( \mathcal{l} \right)},$$

$$\mathrm{FN}_{\mathcal{l}}=\sum_{p} \mathcal{K}_{p\mathcal{,l}}\cdot y_{p}^{\left( \mathcal{l} \right)}\cdot\left( 1-\hat{y}_{p}^{\left( \mathcal{l} \right)} \right), \mathrm{TN}_{\mathcal{l}}=\sum_{p} \mathcal{K}_{p\mathcal{,l}}\cdot\left( 1-y_{p}^{\left( \mathcal{l} \right)} \right)\cdot\left( 1-\hat{y}_{p}^{\left( \mathcal{l} \right)} \right),$$

with derived precision $\mathrm{TP}_{\mathcal{l}}/\left( \mathrm{TP}_{\mathcal{l}}+\mathrm{FP}_{\mathcal{l}} \right)$, recall $\mathrm{TP}_{\mathcal{l}}/\left( \mathrm{TP}_{\mathcal{l}}+\mathrm{FN}_{\mathcal{l}} \right)$, F1 score $2\mathrm{TP}_{\mathcal{l}}/\left( 2\mathrm{TP}_{\mathcal{l}}+\mathrm{FP}_{\mathcal{l}}+\mathrm{FN}_{\mathcal{l}} \right)$ and support $\mathrm{TP}_{\mathcal{l}}+\mathrm{FN}_{\mathcal{l}}$ (positives) and $\mathrm{TP}_{\mathcal{l}}+\mathrm{FN}_{\mathcal{l}}+\mathrm{TN}_{\mathcal{l}}+\mathrm{FP}_{\mathcal{l}}$ (evaluable pairs).

**Inter-rater agreement.** For two raters $r_{1},r_{2}$ on a label $\mathcal{l}$ with $\left| \mathcal{P} \right|$ patients, Cohen’s kappa is

$$\kappa=\frac{p_{o}-p_{e}}{1-p_{e}},$$

with observed agreement $p_{o}=\frac{1}{\left| \mathcal{P} \right|}\sum_{p} \mathbf{1}\left[ y_{p,r_{1}}^{\left( \mathcal{l} \right)}=y_{p,r_{2}}^{\left( \mathcal{l} \right)} \right]$ and chance agreement

$$p_{e}=\sum_{c\in\{0,1\}} \pi_{c,r_{1}}\pi_{c,r_{2}}, \pi_{c,r}=\frac{1}{\left| \mathcal{P} \right|}\sum_{p} \mathbf{1}\left[ y_{p,r}^{\left( \mathcal{l} \right)}=c \right].$$

For $R\geq3$ raters, Fleiss’ kappa is computed as

$$\kappa_{\text{Fleiss}}=\frac{\bar{P}-\bar{P}_{e}}{1-\bar{P}_{e}},$$

with

$$\bar{P}=\frac{1}{\left| \mathcal{P} \right|}\sum_{p} \frac{1}{R\left( R-1 \right)}\sum_{c\in\{0,1\}} n_{p,c}\left( n_{p,c}-1 \right), \bar{P}_{e}=\sum_{c\in\{0,1\}} \pi_{c}^{2},$$

where $n_{p,c}$ is the number of raters who assigned category $c$ to patient $p$ and $\pi_{c}=\frac{1}{\left| \mathcal{P} \right|R}\sum_{p} n_{p,c}$ is the marginal frequency of category $c$. Raw agreement (proportion of identical pairs across all rater pairs) is also reported as a complement to chance-corrected statistics, particularly for low-prevalence labels where $\kappa$ is unstable.

## 1.16 Implementation details

**Software.** All steps are implemented in Python ≥ 3.10. Key packages: ultralytics (YOLOv8x-Pose-P6 inference); tabicl (TabICLv2 classifier; checkpoint tabicl-classifier-v2-20260212.ckpt); pandas, numpy, scipy.stats, sklearn.model_selection.GroupKFold. Pose extraction was performed on Google Colab with a CUDA-enabled GPU; tabular training, tuning, calibration and evaluation were performed on CPU or GPU depending on availability.

**Storage and naming conventions.** Per-window features are cached as gzip-compressed CSV files named features_windows_train__fps{fps}__rn{rn}__post{post}.csv.gz, with rn = 1 if robust normalization was applied (1.7) and post = 1 if posture-centric signals were added (1.4). Per-label trained models are stored under models/label_<phenomenology>/model.joblib. Calibrated artifacts are stored as agg_map.<dataset>.json and thresholds.<dataset>.json.

**Reproducibility.** All random operations (negative subsampling, fold construction, calibration tie-breaking) use a fixed seed (seed = 42). Fold disjointness at the patient level is asserted at runtime. The full code base required to reproduce preprocessing, feature extraction, training, tuning, inference and calibration is released alongside this manuscript at the URL provided in the main text.

**Data.** Original clinical videos cannot be shared because of patient privacy. The derived feature tables and the JSON ground-truth files corresponding to the four reference-label schemes (majority, agreement_aware, strict-k, masked) are released to enable independent re-evaluation of the calibration and decision steps without access to the raw videos.
